# Supplementary material for: Socioeconomic inequalities in young adulthood disrupt the immune transcriptomic landscape via upstream regulators
Source: Res Sq. 2023 Sep 5:rs.3.rs-3295746. Preprint. [Version 1] doi: 10.21203/rs.3.rs-3295746/v1 (PMC10503859; doi:10.21203/rs.3.rs-3295746/v1)
Supplement: Supplement 1 [file NIHPPrs3295746v1-supplement-1.pdf]

Supplementary Files

This is a list of supplementary files associated with this preprint. Click to download.

- SupplementaryDatasetS1.xlsx
- SupplementaryDatasetS2.xlsx
- SupplementaryDatasetS3.xlsx
- SupplementaryInformation.docx
